# Supplementary material for: Institutionalization of limited obstetric ultrasound leading to increased antenatal, skilled delivery, and postnatal service utilization in three regions of Ethiopia: A pre-post study
Source: PLoS One. 2023 Feb 15;18(2):e0281626. doi: 10.1371/journal.pone.0281626 (PMC10045583; doi:10.1371/journal.pone.0281626)
Supplement: S2 File — (DOCX) [file pone.0281626.s002.docx]

**S2 File 2**

| **Tests of Normality** | | | | | | |
| --- | --- | --- | --- | --- | --- | --- |
|  | Kolmogorov-Smirnov^a^ | | | Shapiro-Wilk | | |
|  | Statistic | df | Sig. | Statistic | df | Sig. |
| PNC percent | .093 | 120 | .012 | .957 | 120 | .001 |
| Delivery percent | .095 | 120 | .009 | .949 | 120 | .000 |
| ANC4_1 percent | .086 | 120 | .031 | .938 | 120 | .000 |
| ANC1_1 percent | .073 | 120 | .179 | .968 | 120 | .006 |
| a. Lilliefors Significance Correction | | | | | | |

Table 1: Comparison of the mean monthly ANC1, ANC4, skilled delivery and PNC service uptake before and after ultrasound services introduction in health centers, USAID Transform: Primary health Care Activity, 2017-2020.

|  | ANC1 percent | ANC4 percent | Skilled birth attendance percent | PNC percent |
| --- | --- | --- | --- | --- |
| Kruskal-Wallis H | 17.090 | 16.243 | 23.595 | 17.795 |
| df | 3 | 3 | 3 | 3 |
| Asymp. Sig. | .001 | .001 | .000 | .000 |
| a. Kruskal Wallis Test | | | | |
| b. Grouping Variable: sample4 | | | | |
