# Supplementary material for: Institutionalization of limited obstetric ultrasound leading to increased antenatal, skilled delivery, and postnatal service utilization in three regions of Ethiopia: A pre-post study
Source: PLoS One. 2023 Feb 15;18(2):e0281626. doi: 10.1371/journal.pone.0281626 (PMC10045583; doi:10.1371/journal.pone.0281626)
Supplement: Attachment — Submitted filename: PONE-D-22-17217_Reviewed.pdf [file pone.0281626.s003.pdf]

PLOS ONE

Institutionalization of limited obstetric ultrasound program leads to increased antenatal, skilled delivery and postnatal service utilization in 3 regions of Ethiopia: a pre-post study

--Manuscript Draft--

**Manuscript Number:**	PONE-D-22-17217
**Article Type:**	Research Article
**Full Title:**	Institutionalization of limited obstetric ultrasound program leads to increased antenatal, skilled delivery and postnatal service utilization in 3 regions of Ethiopia: a pre-post study
**Short Title:**	Increased ANC1, ANC4, SBA and PNC service utilization in Ethiopia
**Corresponding Author:**	Mesele Damte Argaw, Ph.D.<br>JSI Research & Training Institute, Inc.,<br>Addis Ababa, ETHIOPIA
**Keywords:**	Ultrasound; institutionalization; ANC; SBA; PNC; limited Obstetric ultrasound; Ethiopia
**Abstract:**	Background: A minimum of one ultrasound scan is recommended for all pregnant women before the 24th week of gestation. In Ethiopia, there is a shortage of skilled manpower to provide these services. Currently, trained mid-level providers are providing the services at the primary healthcare level.<br>Methods: A pre and post intervention observational study was conducted to investigate maternal and neonatal health service utilization rates before and after institutionalizing Vscan limited obstetric ultrasound services, between 2017 and 2020. The data were extracted from 1st August – 31st December 2020.<br>Results: The observed monthly increase on the mean rank of first antenatal care (ANC) visits after the introduction of Vscan limited obstetric ultrasound services showed a statistically significant difference at KW-ANOVA H (3) =17.09, P=0.001. The mean rank of fourth ANC utilization showed a statistically significant difference at KW- ANOVA H (3) =16.24, P=0.001. The observed mean rank in skilled birth attendance (SBA) showed a statistically significant positive difference using KW-ANOVA H (3) =23.6, P<0.001. The mean rank of increased utilization in postnatal care showed a statistically significant difference using KW-ANOVA H (3) =17.79, P<0.001.<br>Conclusion: The introduction of limited obstetric ultrasound services by trained mid-level providers at the primary healthcare level was found to have improved the utilization of ANC, SBA, and postnatal care services. It is recommended that the institutionalization of limited obstetric ultrasound services be scaled up, and a further comparative study between facilities with and without ultrasound services be conducted to confirm causality and assess effects on maternal and perinatal outcomes.
**Order of Authors:**	Hailemariam Segni Abawollo, MD, MPH
	Mesele Damte Argaw, Ph.D.
	Zergu Taffesse Tsegaye, MPH
	Ismael Ali Beshir, MPH
	Asfaw Adugna Guteta, MPH
	Atrie Fekadu Heyi, MPH
	Birhan Tenaw Mengesha, MPH
	Tsega Teferi Mamo, MPH
	Zenawork Kassa Gebremedhin, MPH
	Heran Demissie Damte, BA
	Mengistu Asnake Kibret, MD, MPH
	Binyam Fekadu Desta, PhD

Powered by Editorial Manager® and ProduXion Manager® from Aries Systems Corporation

Additional Information:	
**Question**	**Response**
**Financial Disclosure**<br><br>Enter a financial disclosure statement that describes the sources of funding for the work included in this submission. Review the submission guidelines for detailed requirements. View published research articles from *PLOS ONE* for specific examples.<br><br>This statement is required for submission and **will appear in the published article** if the submission is accepted. Please make sure it is accurate.<br><br>**Unfunded studies**<br>Enter: *The author(s) received no specific funding for this work.*<br><br>**Funded studies**<br>Enter a statement with the following details:<br>• Initials of the authors who received each award<br>• Grant numbers awarded to each author<br>• The full name of each funder<br>• URL of each funder website<br>• Did the sponsors or funders play any role in the study design, data collection and analysis, decision to publish, or preparation of the manuscript?<br>• **NO** - Include this sentence at the end of your statement: *The funders had no role in study design, data collection and analysis, decision to publish, or preparation of the manuscript.*<br>• **YES** - Specify the role(s) played.<br><br>* typeset	This study was fully funded by USAID as part of USAID Transform: Primary Health Care project, implemented under cooperative agreement number of AID-663-A-17-00002 in Ethiopia. The funding body had no role in the design, data collection, analysis, interpretation, and writing stages of the study's undertaking.
**Competing Interests**<br><br>Use the instructions below to enter a competing interest statement for this submission. On behalf of all authors, disclose any competing interests that could be perceived to bias this work—acknowledging all financial support and any other relevant financial or non-financial competing interests.	The authors have declared that no competing interests exist.

Powered by Editorial Manager® and ProduXion Manager® from Aries Systems Corporation

This statement is required for submission and **will appear in the published article** if the submission is accepted. Please make sure it is accurate and that any funding sources listed in your Funding Information later in the submission form are also declared in your Financial Disclosure statement.<br><br>View published research articles from *PLOS ONE* for specific examples.<br><br>**NO authors have competing interests**<br><br>Enter: *The authors have declared that no competing interests exist.*<br><br>**Authors with competing interests**<br><br>Enter competing interest details beginning with this statement:<br><br>*I have read the journal's policy and the authors of this manuscript have the following competing interests: [insert competing interests here]*<br><br>* typeset	
**Ethics Statement**<br><br>Enter an ethics statement for this submission. This statement is required if the study involved:<br><br>• Human participants<br>• Human specimens or tissue<br>• Vertebrate animals or cephalopods<br>• Vertebrate embryos or tissues<br>• Field research<br><br>Write "N/A" if the submission does not require an ethics statement.<br><br>General guidance is provided below. Consult the submission guidelines for detailed instructions. **Make sure that all information entered here is included in the**	This study protocol was carried out in accordance with the Declaration of Helsinki [29] and ethical clearance was granted from the JSI , Institutional Review Board (IRB), reference number IRB#20-26E. The IRB determined that this research activity is exempted from human subject oversight. Informed individual written consent was obtained from each health center manager to extract prenatal care information from routine health information system database. All pregnant mothers personal and medical record information were de-identified using ANC record number. Informed written consent was obtained from all participants. In addition, personal identifiers of pregnant mothers were not captured. The limited obstetric ultrasound scanning program was designed without fetal gender determination for ethical reason and checked regularly by mentors. The investigators maintained national and international ethical principles including ensuring the anonymity and confidentiality of research participants and collected data throughout the research process.

Powered by Editorial Manager® and ProduXion Manager® from Aries Systems Corporation

**Methods section of the manuscript.**<br><br>**Format for specific study types**<br><br>**Human Subject Research (involving human participants and/or tissue)**<br>• Give the name of the institutional review board or ethics committee that approved the study<br>• Include the approval number and/or a statement indicating approval of this research<br>• Indicate the form of consent obtained (written/oral) or the reason that consent was not obtained (e.g. the data were analyzed anonymously)<br><br>**Animal Research (involving vertebrate animals, embryos or tissues)**<br>• Provide the name of the Institutional Animal Care and Use Committee (IACUC) or other relevant ethics board that reviewed the study protocol, and indicate whether they approved this research or granted a formal waiver of ethical approval<br>• Include an approval number if one was obtained<br>• If the study involved *non-human primates*, add *additional details* about animal welfare and steps taken to ameliorate suffering<br>• If anesthesia, euthanasia, or any kind of animal sacrifice is part of the study, include briefly which substances and/or methods were applied<br><br>**Field Research**<br><br>Include the following details if this study involves the collection of plant, animal, or other materials from a natural setting:<br>• Field permit number<br>• Name of the institution or relevant body that granted permission	
**Data Availability**<br><br>Authors are required to make all data underlying the findings described fully available, without restriction, and from the time of publication. PLOS allows rare exceptions to address legal and ethical concerns. See the PLOS Data Policy and FAQ for detailed information.	Yes - all data are fully available without restriction

A Data Availability Statement describing where the data can be found is required at submission. Your answers to this question constitute the Data Availability Statement and **will be published in the article**, if accepted.<br><br>**Important:** Stating 'data available on request from the author' is not sufficient. If your data are only available upon request, select 'No' for the first question and explain your exceptional situation in the text box.<br><br>Do the authors confirm that all data underlying the findings described in their manuscript are fully available without restriction?	
**Describe where the data may be found in full sentences. If you are copying our sample text, replace any instances of XXX with the appropriate details.**<br><br>• If the data are **held or will be held in a public repository**, include URLs, accession numbers or DOIs. If this information will only be available after acceptance, indicate this by ticking the box below. For example: *All XXX files are available from the XXX database (accession number(s) XXX, XXX.*).<br>• If the data are all contained **within the manuscript and/or Supporting Information files**, enter the following: *All relevant data are within the manuscript and its Supporting Information files.*<br>• If neither of these applies but you are able to provide **details of access elsewhere**, with or without limitations, please do so. For example:<br><br>*Data cannot be shared publicly because of [XXX]. Data are available from the XXX Institutional Data Access / Ethics Committee (contact via XXX) for researchers who meet the criteria for access to confidential data.*<br><br>*The data underlying the results*	The data used for this manuscript is attached as supplement file.

presented in the study are available from (include the name of the third party and contact information or URL).<br>• This text is appropriate if the data are owned by a third party and authors do not have permission to share the data.<br><br>* typeset	
Additional data availability information:	Tick here if the URLs/accession numbers/DOIs will be available only after acceptance of the manuscript for publication so that we can ensure their inclusion before publication.

Manuscript Click here to access/download;Manuscript;Manuscript.docx

Institutionalization of limited obstetric ultrasound program leads to increased antenatal, skilled delivery and postnatal service utilization in 3 regions of Ethiopia: a pre-post study

Hailemariam Segni Abawollo [1#a, &], Mesele Damte Argaw[1,#a,&,*], Zergu Taffesse Tsegaye[1, #a, &], Ismael Ali Beshir[1,#a, &], Asfaw Adugna Guteta [1,#a,¶], Atrie Fekadu Heyi[1, #a, ¶], Birhan Tenaw Mengesha[1,#a, ¶], Tsega Teferi Mamo[1,#a, ¶], Zenawork Kassa Gebremedhin[1, #a,¶], Heran Demissie Damte[1#a, ¶], Mengistu Asnake Kibret[2,#b, ¶], Binyam Fekadu Desta[1#a, &]

[1]USAID Transform: Primary Health Care Activity, JSI Research & Training Institute Inc., Ethiopia.

[2]USAID Transform: Primary Health Care Activity, Pathfinder International, Ethiopia.

[#a]Current Address: USAID Transform: Primary Health Care, JSI Training & Research Institute, Inc., Addis Ababa, Ethiopia

[#b]Current Address: USAID Transform: Primary Health Care, Pathfinder International, Addis Ababa, Ethiopia

***Corresponding author**

Email: mdamte5@gmail.com (MDA)

[¶]These authors contributed equally to this work.

[&]These authors also contributed equally to this work.

Abstract

Background: A minimum of one ultrasound scan is recommended for all pregnant women before the 24th week of gestation. In Ethiopia, there is a shortage of skilled manpower to provide these services. Currently, trained mid-level providers are providing the services at the primary healthcare level.

Methods: A pre and post intervention observational study was conducted to investigate maternal and neonatal health service utilization rates before and after institutionalizing Vscan limited obstetric ultrasound services, between 2017 and 2020. The data were extracted from 1st August – 31st December 2020.

Results: The observed monthly increase on the mean rank of first antenatal care (ANC) visits after the introduction of Vscan limited obstetric ultrasound services showed a statistically significant difference at KW-ANOVA H (3) =17.09, P=0.001. The mean rank of fourth ANC utilization showed a statistically significant difference at KW- ANOVA H (3) =16.24, P=0.001. The observed mean rank in skilled birth attendance (SBA) showed a statistically significant positive difference using KW-ANOVA H (3) =23.6, P<0.001. The mean rank of increased utilization in postnatal care showed a statistically significant difference using KW-ANOVA H (3) =17.79, P<0.001.

Conclusion: The introduction of limited obstetric ultrasound services by trained mid-level providers at the primary healthcare level was found to have improved the utilization of ANC, SBA, and postnatal care services. It is recommended that the institutionalization of limited obstetric ultrasound services be scaled up, and a further comparative study between facilities with and without ultrasound services be conducted to confirm causality and assess effects on maternal and perinatal outcomes.

Key words: *Ultrasound, institutionalization, ANC, SBA, PNC limited Obstetric ultrasound, Ethiopia*

Background

The United Nations (UN) member countries have pledged to reduce maternal mortality ratios by at least two thirds by the year 2030 from their 2010 baseline. Even sub-Saharan countries, with heavier burdens, are expected to achieve greater reductions. Similarly, for the same period, counties have targeted to reduce neonatal mortality ratios to as low as 12 per l,000 live births [1]. The stated ambitious commitments of states were supplemented with proven maternal and neonatal health services. These services are offered to women during pregnancy, childbirth, and postpartum periods, whereas a healthy neonatal period starts from 22 completed weeks of gestation continues to the first month of life [2].

Despite the increasing efforts to address challenges, around 40 percent of fetal, neonatal, and maternal deaths occur during the intrapartum period or on the day of birth [3]. Early identification, confirmation, and arrangement of referral services for high-risk health conditions reduces morbidities and mortalities in low and middle-income countries [4] [5]. These conditions all rely on offering at least one ultrasound scanning service for every pregnant woman before the 24th week of gestation for confirming the diagnoses [6]. However, access to ultrasound services is limited in low-resource settings due to human resource constraints and lack of availability of the required technology [7]. Optimizing the capacity of existing human resources and distribution of tasks and responsibilities among various health professionals will improve access to, and quality and equity of health services [8].

During the year 2020, there were 95 radiologists and 462 obstetricians/gynecologists available to provide obstetric ultrasound services in Ethiopia, with a population of over 110 million. In addition, most of the antenatal care (ANC), skilled birth attendance (SBA) i.e. institutional

delivery, and postnatal care (PNC) services are carried out in rural health centers where ultrasound services are not available.

Studies have documented improvements in maternal and neonatal health service utilization and favorable pregnancy outcomes for both mother and baby, as a result of availing obstetric point of care ultrasound services and improved quality of services in remote places around the world [9–20]. The United States Agency for International Development (USAID) Transform: Primary Health Care project has institutionalized antenatal limited obstetric ultrasound services in 100 health centers in Ethiopia. In addition, the Ethiopian ministry of health (MoH) has developed a plan to scale up obstetric ultrasound services in about one thousand health centers by 2025 [21]. Despite this, the comparison ANC1, ANC4, SBA, and PNC services utilizations based on institutionalizing limited obstetric ultrasounds program have not been assessed. Therefore, this study aims to compare ANC1, ANC4, SBA, and PNC service utilization before and after compare institutionalizing Vscan limited obstetric ultrasound program at semi-urban health centers in Ethiopia. To the authors' knowledge, this study is the first of its kind in the country and will be used to guide policy on the area.

Operational definitions:

Institutionalization of limited obstetric ultrasound services: the USAID Transform: Primary Health Care project implemented a step-by-step process of institutionalizing limited obstetric ultrasound services at health center level. The processes followed consisted of laying down the legal framework, capacity building service initiation, and transitioning to the public health sector. A detailed description of the four implemented steps is presented below.

- The first step was working on the legal framework and developing governing documents as a foundation of institutionalizing ultrasound services. Therefore, the project organized a series of preliminary discussions with policy makers, program managers and healthcare providers. The output of these discussions was identification of the roles and responsibilities of stakeholders, development of course syllabus, and identification of the 100 health centers and mapping out of their referral networks.
- The second step implemented was capacity building of mid-level health professionals on operating limited obstetric ultrasound scanning, diagnosis, and management services; orientation of community health workers on promoting health literacy of pregnant mothers, and preparation of service sites with rooms, supplies, and equipment.
- The third step implemented was initiation of antenatal ultrasound services in the 100 selected health centers. In addition, the project maintained and ensured the quality of services through mentoring, coaching, and conducting supervisions.
- The fourth step was enabling a full transition to the public health system. The project trained at least two mid-level health professionals per health center and conducted implementation research on barriers and facilitators of ultrasound services. Using the results, the project advocated for continuous refilling of supplies, training of more providers, and scaling up at new sites using public sector resources.

Methods

Design, period, and setting

A pre- and post-intervention observational study was conducted to understand maternal and neonatal health service utilization rates before and after institutionalizing Vscan limited obstetric ultrasound services between 2017 and 2020. The actual data extraction was made from 1st August

to 31st December 2020. The study was conducted in Amhara, Oromia, and Southern Nations, Nationalities and Peoples' (SNNP) regions. These regions were selected due to the presence of Vscan limited obstetric ultrasound services in selected health centers, for over two years. A health center is a public health facility serving 25,000 inhabitants within the primary healthcare system of Ethiopia which has a mandate to provide promotive, preventive, curative, and rehabilitative outpatient care including basic laboratory and pharmacy services with a capacity for 10 beds for emergency and delivery services [22].

Intervention

USAID funds the USAID Transform: Primary Health Care project (2017- 2022) to support the MoH in line with its long-term goal of preventing child and maternal deaths (PCMD) [23]. To improve access to, and quality and equity of basic maternal and neonatal health services, the project, in collaboration with its technology partner GE Healthcare introduced Vscan access—a small portable ultrasound device, for obstetric scanning by trained mid-level healthcare providers [24]. The project strategizes to increase SBA through introducing ANC limited obstetric ultrasound services and improving the proper management of identified complications of pregnancies in referral health facilities, all of which contribute to maternal, fetal, and neonatal positive health outcomes [23, 25].

One hundred ultrasound machines with seed supplies were provided to each targeted health center, after successfully providing basic limited ultrasound classroom and practical hands-on training for 10 days. The trainees were 219 mid-level healthcare providers, who lacked knowledge and skills on the technology [26]. In addition, the training was supplemented with three sessions of monthly coaching, each lasting for two days and the provision of virtual real-time feedback. Service initiation was supported through awareness creation and dissemination of information on

ultrasound service availability at the health center level using all community engagement platforms including pregnant women conferences, women development army meetings, and house to house visits by health extension workers [23]. To ensure the quality of ultrasound services, a continuous mentorship was carried out by government and project staff. Pregnant women with detected abnormalities during scanning were referred to nearby hospitals for confirmation of the diagnosis and further care. The number of mothers that received ultrasound scanning services for the first, second, and third or more time within the two years after the introduction of the services were 10,186, 2,974, and 1,509, respectively.

Sample size and sampling

The sample size was determined using the rule of thumb recommendation of The Aga Khan Foundation (1997) [27]. Thirty health centers were sampled (30% of the 100 health centers). The three regions were selected based on the accessibility and functionality of the ultrasound services during the time of data collection. Finally, a simple random sampling technique was applied to identify individual facilities.

Data collection

Three supervisors and 30 data collectors who are health science professionals were recruited from the targeted three regions. A two-day training on the objectives of the study, data collection techniques, ethical principles, and field pretesting was carried out. The data were extracted from a routine health information management system (HMIS) database using a pre-tested tool. To ensure the quality of data, three trained supervisors with master's degrees in public health were deployed in the field and provided close technical support, with feedback given daily to the data collectors.

The supervisors were responsible for checking and rechecking the collected data for completeness and consistencies.

The **dependent variables** were summary aggregated continuous data [28] of ANC1, ANC4, SBA, and PNC service reports.

The independent variables were years of service.

Data analysis

The data were entered and cleaned using Microsoft Excel 2016 and exported to SPSS V25 for descriptive and inferential analysis. The service utilization coverages were compared based on institutionalizing Vscan limited obstetric ultrasound program were determined using 1,440 aggregate data collected from the 30 health centers (S1 File 1). In addition, for this study, the steps and procedures of Ross et al., (2013) were adopted [10]. The results of the statistical tests were presented using tables and graphs. To analyze F tests (one-way analyses of variance, ANOVA), the data violated the assumption of homogeneity of variances of a parametric test using Shapiro-Wilk test of normality $P<0.05$ **(S2 File 2)**. Hence, an equivalent non-parametric test called The Kruskal-Wallis H test or a ‘one-way ANOVA on ranks’ was employed to determine statistically significant differences between four groups of independent variables i.e., years of service. The statistical differences were claimed at $P<0.05$. However, the investigators maintained and ensured the following assumptions of Kruskal-Wallis H tests: (1) the dependent variables (ANC, SBA, and PNC) are interval data, (2) the independent variable has four categories, (3) there was no relationship between observations in each group, and (4) the distribution of scores in each group were not identical [28]. Finally, a statistical analysis was employed to compare data from twenty-four months (2017 and 2018) preceding the introduction of Vscan limited obstetric ultrasound

services and twenty-four months (2019 and 2020) following the intervention. The mean rank monthly first ANC, fourth ANC, SBA, and PNC were compared before and after ultrasound services were initiated using a nonparametric test called the Kruskal-Wallis H test.

Ethical considerations

This study protocol was carried out in accordance with the Declaration of Helsinki [29] and ethical clearance was granted from the JSI , Institutional Review Board (IRB), reference number IRB#20-26E. The IRB determined that this research activity is exempted from human subject oversight. Informed individual written consent was obtained from each health center manager to extract prenatal care information from routine health information system database. All pregnant mothers personal and medical record information were de-identified using ANC record number. Informed written consent was obtained from all participants. In addition, personal identifiers of pregnant mothers were not captured. The limited obstetric ultrasound scanning program was designed without fetal gender determination for ethical reason and checked regularly by mentors. The investigators maintained national and international ethical principles including ensuring the anonymity and confidentiality of research participants and collected data throughout the research process.

Results

Description of the study population

Characteristics of the study area and population categorized by region and service years are presented in table 1. The majority (13/30) of the health centers were enrolled from within the Oromia Region. On average, each health center is located 58.6 kilometers away from a referral receiving hospital. At the endpoint, there were about 1.19 million people living within the study areas. Of these, the estimated number of pregnant women eligible for maternal and neonatal health services for the year 2020 were 40,506 (3.4%) (table 1).

Table 1. Characteristics of the study area and population, USAID Transform: Primary health Care project intervention sites, Ethiopia, 2017- 2020.

Characteristics	Amhara	Oromia	SNNP	Total
Number of health centers	9	13	8	30
Average distance from a referral receiving facility in kilometers	59.5	70.4	38.4	58.6
Population				
2017	329,038	529,008	242,815	1,100,861
2018	337,822	543,129	249,298	1,130,249
2019	346,841	557,628	255,953	1,160,422
2020	356,100	572,513	262,785	1,191,398
Eligible women				
2017	11,873	19,270	8,858	40,001
2018	11,949	19,333	8,884	40,166
2019	12,028	19,398	8,909	40,335
2020	12,106	19,464	8,936	40,506

Maternal and neonatal health service utilization

A Kruskal-Wallis test was conducted to determine if there were differences in first ANC, fourth ANC, SBA, and PNC service scores between groups that differed in service years. The adjusted p-values are presented. Figure 1 and table 2 depict the results of a retrospective assessment of maternal and neonatal health service beneficiaries and coverages with a category of prior to and post the introduction of ultrasound services in the 30 health centers.

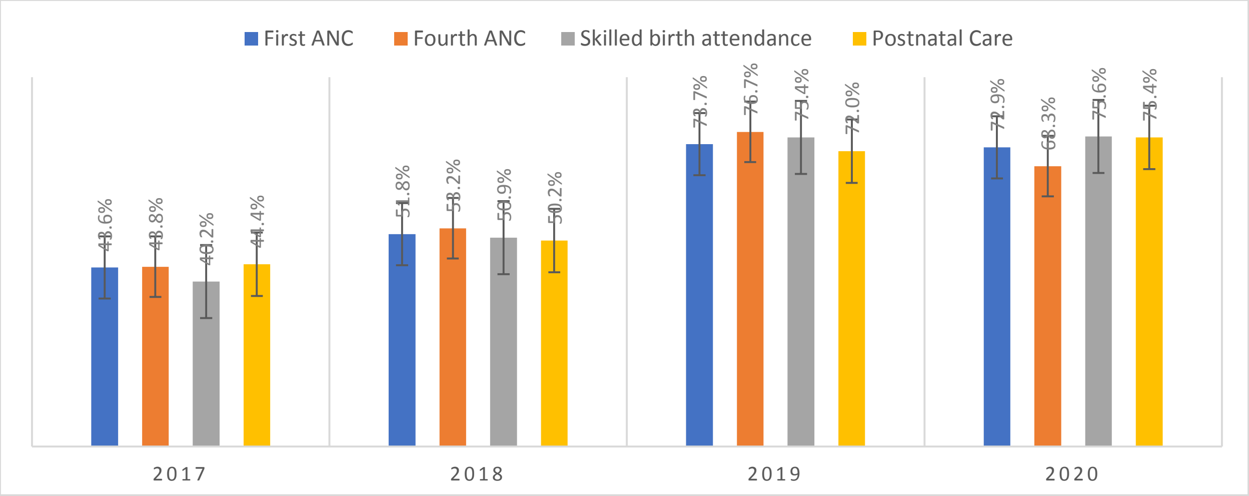

Figure 1. Bar chart showing first ANC, fourth ANC, SBA, and PNC service utilization coverages prior to, and post the introduction of ultrasound services at health centers, Ethiopia, 2017-2020.

The coverages of service utilization for first ANC were 43.6 % and 72.9 %, for the years 2017 and 2020, respectively. In addition, the mean numbers of the first ANC visits were 765 (±SD) and 1126.9 (±SD), for equal reporting periods of pre- and post-ultrasound introduction in the 30 selected health centers. The observed monthly increase on mean rank of first ANC visits after the introduction of Vscan limited obstetric ultrasound service shows a statistically significant difference at KW-ANOVA H (3) = 17.09, P=0.001.

The average fourth ANC visit coverages were 43.8% and 68.3%, for the years 2017 and 2020, respectively. Similarly, the mean numbers of fourth ANC visits were 534 (±SD) and 777.5 (±SD), during the pre- and post-ultrasound introduction periods, respectively. The mean rank of fourth ANC increased utilization shows a statistically significant difference at KW- ANOVA H (3) = 16.24, P=0.001.

The average SBA service utilization coverages were 40.2% and 75.6%, for the years 2017 and 2020 respectively. The mean numbers of SBA in the thirty health centers were 487.9 (±SD) and 761.5 (±SD), twenty-four months pre- and twenty-four months post-ultrasound introduction,

respectively. The observed mean rank SBA has shown a statistically significant positive difference using KW-ANOVA H (3) = 23.6, P<0.001.

The average PNC service coverages were 44.4% and 75.4%, for the years 2017 and 2020, respectively. The mean numbers of PNC service utilization for 48 months in the 30 health centers were 511.8 (±SD) and 790.1(±SD), prior to, and after the introduction of ultrasound services, respectively. The mean rank of PNC service utilization increase has shown a statistically significant difference using KW-ANOVA H (3) = 17.79, P<0.001.

Table 2. Number of monthly ANC, SBA, and PNC services utilization in 30 health centers pre- and post-ultrasound introduction in Ethiopia, 2017-2020.

Measurements	Time	Facility-months	Mean (SD)	Median	Min-Max	95%CI
**Number of first ANC visits**	2017	720	705.7 (378.8)	636.5	136 - 1859	564.3 - 847.2
	2018	720	824.3 (512.2)	625.0	251 - 2253	633.1 - 1015.6
	Pre-ultrasound introduction	1440	765 (450.6)	625.0	136 - 2253	648.6 - 881.4
	2019	720	1130.3 (617.6)	905.5	254 - 2916	899.6 - 1360.9
	2020	720	1123.6 (625.7)	905.5	254 - 2916	889.9 - 1357.3
	Post-ultrasound introduction	1440	1126.9 (616.5)	905.5	254 - 2916	967.7 - 1286.2
**Number of fourth ANC visits**	2017	720	475.7 (243.8)	454.5	126 - 964	384.7 - 566.8
	2018	720	592.3 (409.4)	455.5	218 - 1955	439.5 - 745.2
	Pre-ultrasound introduction	1440	534 (339.2)	454.5	126 - 1955	446.4 - 621.6
	2019	720	802.9 (378.5)	754.0	177 - 2007	661.6 - 944.3
	2020	720	752.1 (431.9)	665.5	148 - 1989	590.8 - 913.4
	Post-ultrasound introduction	1440	777.5 (403.4)	715.0	148 - 2007	673.3 - 881.7
**Number of SBA**	2017	720	423.7 (224.0)	377.5	116 - 964	340.0 - 507.4
	2018	720	552.0 (384.8)	437.0	218 - 1756	408.3 - 695.7
	Pre-ultrasound introduction	1440	487.9 (318.8)	393.5	116 - 1756	405.5 - 570.2
	2019	720	747.6 (320.8)	679.5	232 - 1810	627.8 - 867.4
	2020	720	775.4 (455.8)	677.5	252 - 2427	605.2 - 945.7
	Post-ultrasound introduction	1440	761.5 (391.0)	679.5	232 - 2427	660.4 - 862.5
**Number of PNC visits**	2017	720	464.3 (248.9)	434.0	99 - 983	371.3 - 557.3
	2018	720	559.4 (410.4)	391.5	123 - 1756	406.2 - 712.7
	Pre-ultrasound introduction	1440	511.8 (339.9)	398.0	99 - 1756	424.0 - 599.6
	2019	720	774.6 (454.6)	652.0	201 - 2339	604.8 - 944.3
	2020	720	805.7 (455.9)	705.5	223 - 2427	635.5 - 975.9
	Post-ultrasound introduction	1440	790.1 (451.6)	675.0	201 - 2427	673.5 - 906.8

Discussion

Every year, an estimated 295,000 mothers die due to pregnancy and childbirth related complications [30]. In addition, 2.4 million newborns die within the first month of life, worldwide [31]. Most of these deaths and related morbidities can be prevented through proven and appropriate screening, diagnosis, referral, and complication management of maternal and neonatal healthcare services [32]. The findings of this study demonstrate that improving the quality of services from pregnancy through to the perinatal period has increased utilization of maternal and neonatal health services. In addition, the information generated helps policy makers, program managers and healthcare workers to institutionalize and scale up limited obstetric ultrasound services in health centers within Ethiopia and other low-income countries.

This study has shown that the introduction of limited obstetric ultrasound services by trained mid-level providers at health centers significantly improved the utilization of first and fourth ANC services. These findings are in alignment with studies conducted elsewhere. A scoping review has shown that the introduction of point of care ultrasound services into routine ANC resulted in higher ANC attendance [33]. A study in Uganda showed that the rate of ANC attendance was higher where portable ultrasounds were advertised, and women can be motivated to attend ANC visits when offered the concrete incentive of seeing their baby [18]. Other studies in Uganda showed that the introduction of a low-cost antenatal ultrasound program at a healthcare clinic in rural Uganda was associated with increases in the monthly mean number of ANC visits and increases in the number of women receiving specific recommended ANC interventions [34]. A study in Tanzania has also shown that the introduction of routine ultrasound scanning during ANC visits significantly increased the percentage of women attending ANC clinics by four times or more [35].

Similarly, the introduction of limited obstetric ultrasound services at health centers by trained mid-level providers was found to have significantly improved the utilization of SBA services at time of delivery and the utilization of PNC services after delivery. These findings are also in alignment with studies conducted in other settings. The above-mentioned study in rural Uganda showed that following the introduction of ultrasound services, significant increases were seen in the number of mean monthly deliveries [34]. The study in Tanzania has also shown that the introduction of a simplified ultrasound scanning technology at the lowest levels of care has an effect of motivating women to select health facility deliveries [35]. A study conducted in Ghana showed that the use of a portable ultrasound scan during ANC increased the number of health facility deliveries [36]. Furthermore, a study in rural Eastern China has shown a statistically significant association between antenatal ultrasound scans and the uptake of cesarean section procedures [37].

Limitations: One of the regions where Vscan access limited obstetric ultrasound services were introduced (Tigray), was not included in the study as services were interrupted due to security reasons. Since this study employed a retrospective observational study design, it has a known limitation in regard to claiming causalities. In addition, all possible confounding factors could not be captured as would have been the case in a prospective, randomized, and controlled trial studies.

Conclusion

Ethiopia is one of the countries in the world with low ANC, SBA, and PNC coverages. The introduction of limited obstetric ultrasound services by trained mid-level providers at health centers was found to have improved the utilization of ANC, SBA, and PNC services. Hence, it is recommended that the institutionalization of limited obstetric ultrasound services by trained mid-level providers at health centers be scaled up. A further comparative study between facilities with

and without ultrasound services to confirm causalities and to assess the effects on maternal and perinatal health outcomes is also recommended.

References

[1] United Nations. Department of Economic and Social Affairs. Sustainable Development. Available online: https://sdgs.un.org/ goals (accessed on 18 March 2022).

[2] World Health Organization, World Health Organization. Reproductive Health, World Health Organization. Department of Reproductive Health. Pregnancy, childbirth, postpartum, and newborn care: a guide for essential practice. World Health Organization; 2003.

[3] Lawn JE, Blencowe H, Oza S, You D, Lee AC, Waiswa P, et al. Every Newborn: progress, priorities, and potential beyond survival. Lancet. 2014 Jul 12; 384:189–205.

[4] Starrs AM. Safe motherhood initiative: 20 years and counting. Lancet. 2006 Sep 30;368(9542):1130–2.

[5] Lee AC, Lawn JE, Cousens S, Kumar V, Osrin D, Bhutta ZA, et al. Linking families and facilities for care at birth: what works to avert intrapartum-related deaths? Int J Gynaecol Obstet. 2009 Oct; 107(Suppl 1): S65–S88.

[6] World Health Organization. WHO recommendations on antenatal care for a positive pregnancy experience. World Health Organization; 2016.

[7] De Stigter K, Pool KL, Leslie A, Hussain S, Tan BS, Donoso-Bach L, Andronikou S. Optimizing integrated imaging service delivery by tier in low-resource health systems. Insights into Imaging. 2021 Dec;12(1):1-1.

[8] World Health Organization. WHO recommendations: optimizing health worker roles to improve access to key maternal and newborn health interventions through task shifting, optimize MNH. World Health Organization; 2012.

[9] Greenwold N, Wallace S, Prost A, Jauniaux E. Implementing an obstetric ultrasound training program in rural Africa. Int J Gynaecol Obstet. 2014 Mar;124(3):274-7. doi: 10.1016/j.ijgo.2013.09.018.

[10] Ross AB, DeStigter KK, Rielly M, Souza S, Morey GE, Nelson M, Silfen EZ, Garra B, Matovu A, Kawooya MG. A low-cost ultrasound program leads to increased antenatal clinic visits and attended deliveries at a health care clinic in rural Uganda. PloS one. 2013;8(10).

[11] Mbuyita S, Tillya R, Godfrey R, Kinyonge I, Shaban J, Mbaruku G. Effects of introducing routinely ultrasound scanning during Ante Natal Care (ANC) clinics on number of visits of ANC and facility delivery: a cohort study. Archives of Public Health. 2015 Dec;73(1):36.

[12] Milart PH, Molina CA, Prieto-Egido I, Martínez-Fernández A. Use of a portable system with ultrasound and blood tests to improve prenatal controls in rural Guatemala. Reproductive health. 2016 Dec 1;13(1):110.

[13] Vinayak S, Sande J, Nisenbaum H, Nolsøe CP. Training midwives to perform basic obstetric point-of-care ultrasound in rural areas using a tablet platform and mobile phone transmission technology—A WFUMB COE project. Ultrasound in medicine & biology. 2017 Oct 1;43(10):2125-32.

[14] Westerway SC. Comparing the effectiveness of training course formats for point- of- care ultrasound in the third trimester of pregnancy. Australasian Journal of Ultrasound in Medicine. 2019 Feb;22(1):45-50.

[15] Wastlund D, Moraitis AA, Dacey A, Sovio U, Wilson EC, Smith GC. Screening for breech presentation using universal late-pregnancy ultrasonography: A prospective cohort study and cost effectiveness analysis. PLoS medicine. 2019 Apr;16(4).

[16] Swanson, J., Kawooya, M., Swanson, D. et al. The diagnostic impact of limited, screening obstetric ultrasound when performed by midwives in rural Uganda. J Perinatol 34, 508–512 (2014). https://doi.org/10.1038/jp.2014.54.

[17] Kozuki N, Mullany LC, Khatry SK, Ghimire RK, Paudel S, Blakemore K, Bird C, Tielsch JM, LeClerq SC, Katz J. Accuracy of home-based ultrasonographic diagnosis of obstetric risk factors by primary-level health workers in rural Nepal. Obstetrics and gynecology. 2016 Sep;128(3):604.

[18] Cherniak W, Anguyo G, Meaney C, Kong LY, Malhame I, Pace R, Sodhi S, Silverman M. Effectiveness of advertising availability of prenatal ultrasound on uptake of antenatal care in rural Uganda: A cluster randomized trial. PloS one. 2017;12(4).

[19] Keable J, Crozier K. Detection of breech presentation: Abdominal palpation and hand-held scanning by midwives. British Journal of Midwifery. 2018 Jun 2;26(6):371-6.

[20] Kawooya MG, Nathan RO, Swanson J, Swanson DL, Namulema E, Ankunda R, Kirumira F, Ddungu-Matovu P. Impact of introducing routine antenatal ultrasound services on reproductive health indicators in Mpigi District, Central Uganda. Ultrasound quarterly. 2015 Dec 1;31(4):285-9.

[21] The Federal Democratic Republic of Ethiopia, Ministry of Health. 2021. Health Sector Transformation Plan II, 2020/21-2024/25. Addis Ababa, Ethiopia.

[22] Ethiopian Standard Agency (ESA). Health Center Requirements. Addis Ababa: Ethiopian Standard Agency; 2011.

[23] USAID Transform: Primary Health Care Project. Theory of change in practice 2017. Addis Ababa: USAID Transform: Primary Health Care; 2017.

[24] GE Healthcare. Vscan AccessTM Ultrasound. Assessing risk. Expanding reach. © 2015 General Electric Company

[25] Franklin, H.L., Mirza, W., Swanson, D.L. et al. Factors influencing referrals for ultrasound-diagnosed complications during prenatal care in five low- and middle-income countries. Reprod Health 15, 204 (2018).

[26] Argaw MD, Abawollo HS, Tsegaye ZT, Beshir IA, Damte HD, Mengesha BT, Gebremedhin ZK, Heyi AF, Guteta AA, Mamo TT, Anara AA. Experiences of midwives on Vscan limited

obstetric ultrasound use: a qualitative exploratory study. BMC Pregnancy and Childbirth. 2022 Dec;22(1):1-2.

[27] The Aga Khan Foundation. Primary Care Advancement Program: assessing the quality of service. 2nd edition. Washington DC.: The Aga Khan Foundation, 1997.

[28] Moineddin R, Urquia ML. Regression analysis of aggregate continuous data. Epidemiology. 2014 Nov 1;25(6):929-30.

[29] World Medical Association. World Medical Association Declaration of Helsinki. Ethical principles for medical research involving human subjects. Bull World Health Organ. 2001;79(4):373.

[30] World Health Organization. Trends in maternal mortality 2000 to 2017: estimates by WHO, UNICEF, UNFPA, World Bank Group and the United Nations Population Division. 2019.

[31] World Health Organization. Newborns: improving survival and well-being. Mediacentre: World Health Organization. Geneva: World Health Organization. 2020.

[32] Gülmezoglu AM, Lawrie TA, Hezelgrave N, Oladapo OT, Souza JP, Gielen M, Lawn JE, Bahl R, Althabe F, Colaci D, Hofmeyr GJ. Interventions to reduce maternal and newborn morbidity and mortality. Reproductive, Maternal, Newborn, and Child Health. 2016 May 27;2:115-36.

[33] Mikaela Doig, Janine Dizon, Katherine Guerrero and Nayana Parange. Exploring the availability and impact of antenatal point-of-care ultrasound services in rural and remote communities: A scoping review. Austarlas J Ultrason Med. August 2019; 22 (3): 174-185.

[34] Andrew B Ross et al. Ancillary benefits of antenatal ultrasound: an association between the introduction of a low-cost ultrasound program and an increase in the numbers of women receiving recommended antenatal treatments. BMC Pregnancy and Childbirth (2014) 14:424. DOI 10.1186/s12884-014-0424-9

[35] Selemani Mbuyita et al. Effects of introducing routinely ultrasound scanning during Ante Natal Care (ANC) clinics on number of visits of ANC and facility delivery: a cohort study. Archives of Public Health (2015) 73:36. DOI 10.1186/s13690-015-0086-8.

[36] Benjamin Amoah et al. Boosting antenatal care attendance and number of hospital deliveries among pregnant women in rural communities: a community initiative in Ghana based on mobile phones applications and portable ultrasound scans. BMC Pregnancy and Childbirth (2016) 16:141. DOI 10.1186/s12884-016-0888-x.

[37] Huang et al. Utilization of antenatal ultrasound scan and implications for caesarean section: a cross sectional study in rural Eastern China. BMC Health Services Research 2012, 12:93. http://www.biomedcentral.com/1472-6963/12/93.

Acknowledgments

The authors would like to acknowledge USAID for funding the study. We are indebted to data managers, data collectors, supervisors, and health facility heads.

Competing interests

The authors declare that they have no competing interests.

Author contributions

Conceptualization: HSA, MDA, ZTT, IAB, BFD

Data curation: . AAG, AFH, BTM, TTM, ZKG

Formal analysis: MDA

Funding acquisition: BFD, MAK

Investigation: HSA MDA

Methodology: HSA, MDA, ZTT, IAB, BFD

Project administration: BFD, MAK

Resources: BFD, MAK

Software: MDA

Supervision: HSA, MDA, ZTT, IAB, BFD

Validation: HDD, BFD, MAK

Visualization: MDA

Writing ± original draft: MDA, HAS, HDD

Writing ± review & editing: HSA, MDA, ZTT, IAB, AAG, AFH, BTM, TTM, ZKG, HDD, MAK, BFD

Funding

This study was fully funded by USAID as part of USAID Transform: Primary Health Care project, implemented under cooperative agreement number of AID-663-A-17-00002 in Ethiopia. The funding body had no role in the design, data collection, analysis, interpretation, and writing stages of the study's undertaking.

Data File

Click here to access/download
Supporting Information
S1 File 1.xlsx

Statistical assumption

Click here to access/download
Supporting Information
S2 File 2.docx
