# Supplementary material for: Institutionalization of limited obstetric ultrasound leading to increased antenatal, skilled delivery, and postnatal service utilization in three regions of Ethiopia: A pre-post study
Source: PLoS One. 2023 Feb 15;18(2):e0281626. doi: 10.1371/journal.pone.0281626 (PMC10045583; doi:10.1371/journal.pone.0281626)
Supplement: Attachment — Submitted filename: Response to reviewers.docx [file pone.0281626.s004.docx]

Reviewer comments and authors responses

Journal requirements

1. Please ensure that your manuscript meets PLOS ONE's style requirements, including those for file naming. The PLOS ONE style templates can be found at
   <https://journals.plos.org/plosone/s/file?id=wjVg/PLOSOne_formatting_sample_main_body.pdf> and 
   <https://journals.plos.org/plosone/s/file?id=ba62/PLOSOne_formatting_sample_title_authors_affiliations.pdf>

Response: Thank you so much for sharing the guides and links. Our manuscript has been corrected accordingly

2. Thank you for stating the following financial disclosure: "This study was fully funded by USAID as part of USAID Transform: Primary Health Care project, implemented under cooperative agreement number of AID-663-A-17-00002 in Ethiopia. The funding body had no role in the design, data collection, analysis, interpretation, and writing stages of the study’s undertaking."

Response: Thank you so much for the comments and suggestions. The Financial disclosure statement is revised as presented below.

This study was fully funded by USAID as part of USAID Transform: Primary Health Care project, implemented by Pathfinder International and JSI Research & Training Institute Inc under cooperative agreement number of AID-663-A-17-00002 in Ethiopia. The project purchased Vscan limited obstetric ultrasound machines and distributed it with seed supplies to 100 health centers. The cost of basic ultrasound training was also covered by the project. In addition, the project covers the cost of data collectors. The funder provided support in the form of salaries for authors HAS, MDA, ZTT, IAB, AAG, AFH, BTM, TTM, ZKG, HDD, MAK, and BFD. However, the funding body had no role in the study design, data collection, analysis, decision to publish, or preparation of the manuscript.

3. Thank you for stating the following in the Acknowledgments Section of your manuscript:
   "The authors would like to acknowledge USAID for funding the study. We are indebted to data managers, data collectors, supervisors, and health facility heads." We note that you have provided funding information that is not currently declared in your Funding Statement. However, funding information should not appear in the Acknowledgments section or other areas of your manuscript.

Response: Thank you so much for the comments and suggestions. The funding information is deleted form acknowledgements and rephrased as follow.

# Acknowledgments

We are indebted to all data managers, data collectors, supervisors, and health facility heads for their meticulous work in this research activity.

4. In your Data Availability statement, you have not specified where the minimal data set underlying the results described in your manuscript can be found. PLOS defines a study's minimal data set as the underlying data used to reach the conclusions drawn in the manuscript and any additional data required to replicate the reported study findings in their entirety. All PLOS journals require that the minimal data set be made fully available. For more information about our data policy, please see <http://journals.plos.org/plosone/s/data-availability>

Response: Thank you so much for the comments. Please update the data availability statement as follow.

All relevant data are within the manuscript and supporting information files.

5. We note that you have stated that you will provide repository information for your data at acceptance. Should your manuscript be accepted for publication, we will hold it until you provide the relevant accession numbers or DOIs necessary to access your data. If you wish to make changes to your Data Availability statement, please describe these changes in your cover letter and we will update your Data Availability statement to reflect the information you provide.

Response: Thank you so much for the comments. Please see the response for Comment # 4 above.

6. Please include captions for your Supporting Information files at the end of your manuscript, and update any in-text citations to match accordingly. Please see our Supporting Information guidelines for more information: <http://journals.plos.org/plosone/s/supporting-information>.

Response: Thank you so much for the comments. All supporting information are presented at the end of the manuscript and in-test citations.

Supplementary information

S1 Fig 1.: Bar chart showing first ANC, fourth ANC, SBA, and PNC service utilization coverages prior to, and post the introduction of ultrasound services at health centers, Ethiopia, 2017-2020.

S1 Table 1.: Characteristics of the study area and population, USAID Transform: Primary health Care project intervention sites, Ethiopia, 2017- 2020.

S2 Table 2.: Number of monthly ANC, SBA, and PNC services utilization in 30 health centers pre- and post-ultrasound introduction in Ethiopia, 2017-2020.

S3 Table 3.: Mean rank scores of ANC1, ANC4, SBA, PNC services and test of difference in its distribution over four years (2017- 2020)

S4 Table 4.: Pairwise comparisons of ANC1 ANC4 Delivery and PNC service coverages over four years (2017 – 2020)

S1 File 1.: Data file

2 File 2.: Statistical assumptions

Summary of the research and overall impression

The reviewers acknowledged the importance of this novel research work and appreciate the effort made to understand the intervention deployed and measured outcomes. The study is very laudable and inculcate the importance of task shifting/sharing to improve quality of services in different point of care.

Discussion on specific area of improvement

Introduction

1. Line 143: Objective - the second objective is to “*develop* malaria care service modalities……” but nothing was developed from the paper, rather than this paper provide input for development of

The wording of the aim of this formative assessment now has been updated. The last paragraph of the introduction, lines 109-112 now read as:

“We conducted a formative assessment to collect baseline data on seasonal migrant workers access to malaria prevention and control interventions. The data generated will provide empirical evidence for the development of malaria care service modalities and elimination strategies in seasonal migrant/mobile workers in Ethiopia.”

Background

2. Page 3, line 63: counties-Correct to countries

Thank you so much for the comment. On page 3, line 63, the word “countries” is corrected.

3. Page 4, line 92: compare-Please delete
   Thank you so much for the comment. On page 4, line 92, the second “compare” is deleted.

Methods

4. Page 5, line 124: Please include the months during which the interventions were carried out. See also page 6, line133.

Thank you so much for the comment. On page 5, line 124, and page 6, line 133 the months of interventions were included.

5. Page 8. line 176: were compared-Please delete

Thank you so much for the comment. On page 8, line 186, the word compare is deleted.

Results

6. Page 10, lines 215-223:
   Comment: The characteristics of the study population should be described in the methodology section and not the result section. Please mention this as a subsection in materials and methods.
   Maternal and neonatal services utilization

Thank you so much for the comment. The study population is moved to methodology section. Please see the changes on page 78, lines 156 – 165.

7. Comment: This section has mainly compared 2017 and 2020 without considering the trend in between these years. The authors should mention the decline in utilization when observed and explanations offered in the discussion section

Thank you so much for the comment. On page 12 lines 256- 257, the mean fourth ANC coverage showed a reduction from 76.68% to 68.32%. This might be due to interruption of services due to stockout of essential supplies and turnover of trained professionals. On page 16, lines 328 – 330, the discussion section included the following information.

However, the trends of ANC4 coverages showed reduction from 2019 to 2020.This might be happed due to interruption of services due to stockout of essential supplies and turnover of trained professionals.
Page 11, lines 223-224:

8. Comment: Please rephrase to: The coverage of first ANC showed an increasing trend from 43.6% in 2017 to 72,9% in 2020

Thank you so much for the comment. The information of maternal and neonatal health services is rephrased. Please see changes on page11 lines 241- 247.

9. Table 2: Please include P-value at the relevant components of the table

Thank you so much for the comment. Table 3 presents the detail information of KW- ANOVA test results. Please see changes on page13 lines 272- 274.

Discussion

10. This section needs to be extensively revised as there was no robust discussion of the four maternal and newborn health services being studied. For example, ANC improved following the intervention apart from comparing with previous studies what is the implication of improved ANC attendance to maternal and newborn health. Is it possible that the benefit of satisfaction gained by the pregnant woman from the new technology encouraged her to deliver in the hospital under the supervision of a SBA attendant and subsequently attend follow up at PNC? Were the ultrasound scan services provided free to the patients?
    The ultrasound scan could have enhanced diagnosis of impending complications and prompt referrals and interventions. How does this align with the overall goal of project? This is a health system strengthening intervention that has shifted a task to breach dearth of human resources for health in Ethiopia, what are the key lessons learnt? Can these lessons be considered in other relevant areas in service delivery in MNCH like in gynecological emergencies for diagnosis of ectopic pregnancies and abortions etc?

Thank you so much for the comment. The discussion section revised, and detail information are included. The following changes are made. Please see changed on page13 lines 294 – 298, 308 – 310, 313- 317, 328- 330.

11. Page 13, lines 259-263
    Comment: These are repetitions from the introduction and not necessary in the discussion section.

Thank you so much for the comment. The first paragraph of the discussion is removed.

12. Page 13, lines 263-267
    Comment: Consider moving this towards the end of the discussion section as this relates to transforming research findings to policy and practice and usually should come after the discussion of findings from the study.

Thank you so much for the comment. The information is moved to the end of the discussion section. On Page 16, lines 325- 330.

Limitation

13. Authors should also consider highlighting in the manuscript if there are other maternal and newborn health interventions by other agencies in these hospital during the study periods that may have affected the quality of care; as change in utilization of MNCH services can be shaped by other sociocultural and community factors which have not been mentioned in this manuscript.

Thank you so much for the comment. The limitation of the study is rephrased. See changes on page 16, lines 330 -336.

14. On a general note, the reviewers have raised concerns in the following areas:

- Statistical analysis and need to include the P-values in table 2
- Description of the study population characteristics under the materials and methods section and not in the result section
- Improvement in the discussion section

Thank you so much for the comment. All the three comments are properly addressed.

Reviewer's Responses to Questions

Comments to the Author

1. Is the manuscript technically sound, and do the data support the conclusions?

The manuscript must describe a technically sound piece of scientific research with data that supports the conclusions. Experiments must have been conducted rigorously, with appropriate controls, replication, and sample sizes. The conclusions must be drawn appropriately based on the data presented.

Reviewer #1: Partly

Reviewer #2: Partly

Thank you so much for the comment. The revised manuscript will satisfy reviewers in the current revised form.

2. Has the statistical analysis been performed appropriately and rigorously?

Reviewer #1: No

Reviewer #2: Yes

Thank you so much for the comment. The statistical test includes the comments of reviewers. We hope, the reviewers will be satisfied in the revised form.

3. Have the authors made all data underlying the findings in their manuscript fully available?

The [PLOS Data policy](http://www.plosone.org/static/policies.action#sharing) requires authors to make all data underlying the findings described in their manuscript fully available without restriction, with rare exception (please refer to the Data Availability Statement in the manuscript PDF file). The data should be provided as part of the manuscript or its supporting information, or deposited to a public repository. For example, in addition to summary statistics, the data points behind means, medians and variance measures should be available. If there are restrictions on publicly sharing data—e.g. participant privacy or use of data from a third party—those must be specified.

Reviewer #1: No

Reviewer #2: Yes

Thank you so much for the comment. All relevant data are within the manuscript and supporting information files.

4. Is the manuscript presented in an intelligible fashion and written in standard English?

PLOS ONE does not copyedit accepted manuscripts, so the language in submitted articles must be clear, correct, and unambiguous. Any typographical or grammatical errors should be corrected at revision, so please note any specific errors here.

Reviewer #1: Yes

Reviewer #2: Yes

Thank you so much for the encouraging comments.

5. Review Comments to the Author

Reviewer #1: REVIEW OF THE ARTICLE


Thank you for asking me to review the above article.

15. The authors have conducted research looking at interventions to reduce maternal mortality by increasing antenatal, skilled delivery and post-natal services. This is a very laudable and ambitious plan. Also inculcating task shifting/sharing with mid-level providers as a part of the protocol is novel and forward thinking

Thank you so much for the encouraging comments.

16. The title is well articulated and self-explanatory.

Thank you so much for the encouraging comments.

17. The authors have chosen to use a structured abstract which is concise and written in very plain language. All aspects of the study are included within the abstract

Thank you so much for the encouraging comments.

18. Line 92 the second ‘compare’ at the end should be expunged

Thank you so much for the comments. The second compare is deleted now.

Methodology#

19. The authors have described their study population, but this should be better placed under methodology

Thank you so much for the comments. The description of the study population is moved to methodology section. Please see the changes on page 78, lines 156 – 165.

20. The sampling technique is appropriate and clearly written and shows how the thirty health centers were chosen, the number of ultrasound machines deployed and how the midlevel staff were all trained.

Thank you so much for the encouraging comments.

21. The Authors have chosen to use an equivalent nonparametric test to ANOVA for their data analyses. The Kruskal Wallis test is appropriate in that regard since more than 2 groups are being analyzed. The groups are segregated by years of service. The Kruskal Wallis test of ranks therefore seeks to define whether there is any difference in the means of the ranked data among the groups. Each group has 4 categories viz ANC1, ANC4, skilled delivery and post-natal care. The presentation of the data is a bit confusing for me, as I would have expected each outcome(ANC1, ANC4, skilled delivery and post-natal care )for the 4 years to have been subjected to the KW test, and any significant difference, or lack thereof would be clearly stated.
    To define which of the years had a more significant difference from the others , a post hoc test would then be applied to showcase this difference.

Thank you so much for the comments. Two tables on Kruskal Wallis H test and post hoc tests are included in the current form.

22. The data was further disaggregated into pre and post introduction of ultrasound. The years 2017 and 2018 represented the years prior to the introduction of the intervention while 2019 and 2020 represented the years after the introduction of said intervention. (Lines 189 to 193). At this point a null and alternative hypothesis would have been nice so as to define exactly the desired outcomes of the study however, having divided the data into 2 independent groups any statistical difference would be explored using the independent sample T test. The authors had correctly stated that this was not a normally distributed sample therefore the corresponding nonparametric test (Mann Whitney U test) might have served better than the Kruskal Wallis test in this instance. The data had already been ranked and as such the rank sum would be a fairly straightforward calculation, with subsequent derivation of the P value using the software specified.

Thank you so much for the comments. Since the authors want to determine distribution of maternal and neonatal health service coverages over four years period, we prefer to work on Kruskal Wallis H test and post hoc tests than dividing the data into two.

23. I would advise the authors should include a table showcasing the rank sum and mean rank per facility. Also, p values should be added where necessary in the various tables

Thank you so much for the comments. Table 3 and 4 have presented the mean rank scores and associated p-values.

24. The discussion is well articulated and the direction of the argument of the authors is clear and well understood
    Thank you so much for the encouraging comments.

Reviewer #2:

25. This is a good manuscript describing the obstetrics outcomes improvement interventions. The manuscript is well written with good layout. However, the discussion section failed to adequately elaborate on the findings of the study. It is suggested that the authors take time to relate the findings with findings from similar studies and relate with the objectives of the process that is being reviewed.

Thank you so much for the encouraging comments.

26. My other comments are as highlighted with information for the attentions of the authors in the added sticky notes.

Which cadre of healthcare workers constitute this mid-level health professionals?

Thank you so much for the comments. The mid-level health professionals are BSc nurses and midwives.

When in 2017 did the implementation of the intervention start and when in 2020 did the intervention end?

Thank you so much for the comments. The study period was July 2016 through June 2020.

What is GE?

Thank you so much for the comments. GE is presented in its expanded form General Electric.

Recast this statement as this read like 100 ultrasound machines were provided to each of the health center!

Thank you so much for the comments. The sentence id rephrased as follow:

”Vscan ultrasound machine with seed supplies were provided to each targeted 100 health centers,”

What accounted for this 3.4 times drop between first and second ultrasound visits?

Thank you so much for the comments. The US scanning services is recommended to be conducted on 13 weeks of gestation to diagnose gross anomalies. And the second scanning services is after 24 weeks to rule out high risk conditions. The significant difference on US 1, US2 and US3 scanning services were in line with the reduction of subsequent ANC service utilization.

Delete the phrase “were determined”

Thank you so much for the comments and the phrase is deleted.

The discussion section needs to be greatly improved on to bring out the findings of this study and compare with similar studies!

Thank you so much for the comments, The discussion is revised and improved in the current form.
